# Low Parasitemia in Submicroscopic Infections Significantly Impacts Malaria Diagnostic Sensitivity in the Highlands of Western Kenya

**DOI:** 10.1371/journal.pone.0121763

**Published:** 2015-03-27

**Authors:** Eugenia Lo, Guofa Zhou, Winny Oo, Yaw Afrane, Andrew Githeko, Guiyun Yan

**Affiliations:** 1 Program in Public Health, College of Health Sciences, University of California at Irvine, Irvine, CA, 92697, United States of America; 2 Centre for Vector Biology and Control Research, Kenya Medical Research Institute, Kisumu, 40100, Kenya; Walter & Eliza Hall Institute, AUSTRALIA

## Abstract

Asymptomatic malaria infections represent a major challenge in malaria control and elimination in Africa. They are reservoirs of malaria parasite that can contribute to disease transmission. Therefore, identification and control of asymptomatic infections are important to make malaria elimination feasible. In this study, we investigated the extent and distribution of asymptomatic malaria in Western Kenya and examined how varying parasitemia affects performance of diagnostic methods including microscopy, conventional PCR, and quantitative PCR. In addition, we compared parasite prevalence rates and parasitemia levels with respect to topography and age in order to explore factors that influence malaria infection. Over 11,000 asymptomatic blood samples from children and adolescents up to 18 years old representing broad areas of Western Kenya were included. Quantitative PCR revealed the highest parasite positive rate among all methods and malaria prevalence in western Kenya varied widely from less than 1% to over 50%. A significantly lower parasitemia was detected in highland than in lowland samples and this contrast was also observed primarily among submicroscopic samples. Although we found no correlation between parasitemia level and age, individuals of younger age group (aged <14) showed significantly higher parasite prevalence. In the lowlands, individuals of aged 5–14 showed significantly higher prevalence than those under age 5. Our findings highlight the need for a more sensitive and time-efficient assay for asymptomatic malaria detection particularly in areas of low-transmission. Combining QPCR with microscopy can enhance the capacity of detecting submicroscopic asymptomatic malaria infections.

## Introduction

Asymptomatic malaria infection is a major obstacle to controlling and eliminating malaria in many African countries. Asymptomatic infections represent reservoirs of malaria parasite that can contribute to resurgence of disease transmission at the right conditions [1, 2]. It is imperative to know the extent and distribution of asymptomatic infections in local communities and to accurately detect infections so as to reduce hidden malaria burden and make malaria elimination feasible. Asymptomatic infections can be caused by both high and low parasite density. However, it is unclear whether the parasitemia level varied across different geographic areas, for example, between highlands and lowlands, and whether such differences in parasitemia influence efficiency and reliability of microscopy and PCR-based diagnostic methods. This is particularly relevant to submicroscopic infections because subtle differences in parasitemia level could influence diagnostic outcomes [3–5]. It is hypothesized that frequent infection in high-transmission areas may increase the average parasite density in infected individuals, whereas in lower transmission areas infections may have reached a submicroscopic phase [6]. However, there is insufficient evidence to support this hypothesis. Moreover, there is ample evidence for peak prevalence in younger age groups for severe [7, 8] and uncomplicated clinical malaria [9], as well as for asymptomatic malaria among different transmission settings [10, 11]. However, it remains unclear whether parasitemia level varies with age given that younger children may exhibit lower parasite tolerance than the older ones.

In areas where malaria endemicity is low control programmes need increasingly sensitive tools for identifying submicroscopic hidden malaria infections and monitoring malaria transmission intensity [1]. Diagnosis of clinical malaria and estimates of asymptomatic malaria prevalence are primarily based on microscopy and rapid diagnostic tests (RDTs) in most malarious areas. Although several previous studies have demonstrated better performance of PCR-based diagnostic methods [12–18], in most healthcare facilities in Africa microscopy remains the standard and simple diagnostic method for symptomatic cases in resource-limited countries or in remote areas where laboratory setting is often lacking [19]. Because interpretation of blood smears often requires considerable expertise, microscopic test can give false results especially when parasitemia level is low (submicroscopic level). PCR, on the other hand, is a less subjective test and has been shown to be more sensitive (detecting 0.5–10 parasites/μL compared to 50–500 parasites/μL by microscope) [12, 13, 20, 21]. Compared to conventional PCR, quantitative real-time PCR (QPCR) method uses fluorescent labels for continuous monitoring of amplicon formation throughout the reaction and provides quantity of parasite DNA copies, which is otherwise impossible by conventional PCR. QPCR may offer more reliable information for infection detection particularly concerning submicroscopic asymptomatic infections [15, 22, 23].

The present study sought to determine (1) the prevalence of asymptomatic *P*. *falciparum* infections in broad areas of western Kenya, (2) whether there is a difference in parasitemia level in asymptomatic infections especially the submicroscopic infections between high-transmission and low-transmission areas as well as among age groups, and (3) whether microscopy and PCR-based approaches give different level of sensitivity and specificity when diagnosing asymptomatic infections at different transmission settings. Characterizing and quantifying parasitemia in asymptomatic infections will provide more in-depth information for malaria control and elimination planning.

## Materials and Methods

### Scientific and ethical statement

Scientific and ethical clearance was given by the institutional scientific and ethical review boards of the Kenya Medical Research Institute, Kenya and the University of California, Irvine, USA. Written informed consent/assent for study participation was obtained from all consenting heads of households, parents/guardians (for minors under age of 18), and each individual who was willing to participate in the study.

### Areas and subjects of study

**Fig 1:**
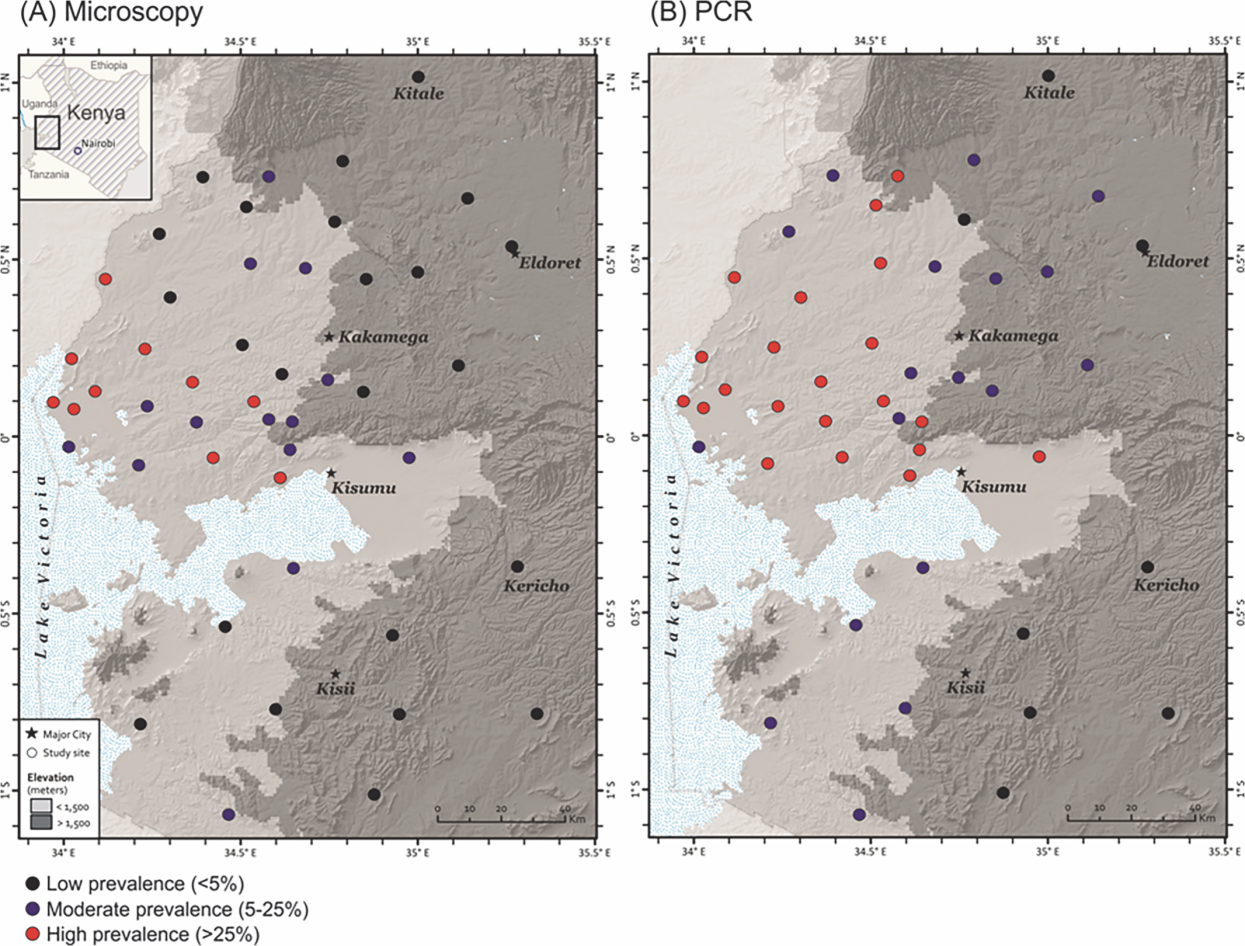
Malaria prevalence of studied sites in western Kenya based on (A) microscopy and (B) conventional PCR. Locality information can be referred to S1 Table. Areas of elevation below 1500 m were indicated by light gray and above 1500 m by dark gray. Black, blue, and red circles represent sites of low (<5%), moderate (5–25%), and high (>25%) malaria prevalence.

Blood samples were collected from a total of 47 sites within a 200×150 km^2^ area of Western Kenya (1°12´N-1°12´S and 34°0´E-35°30´E; Fig. 1; S1 Table). This area encompasses an elevation gradient from ca.1,100m in the Lake Victoria basin to over 2,500m in the highland zone west of the Great Rift Valley. Sampling sites were selected every 20 km from the lakeshore lowland area (defined as an elevation range of 1,000–1,500 m; Fig. 1; S1 Table) towards the northeastern and southeastern slopes of the highland area (defined as an elevation range of 1,500–2,500 m). Fingerprick blood samples were collected from 200–300 schoolchildren aged 3 to 18 years old in each site. Altogether more than 11,000 individuals were examined during June-August, 2011 and 2012. Individuals included in this study showed no fever or malaria-related symptoms at the time of sampling. Thick and thin blood smears were prepared for microscopic examination and 30–50μl of blood was blotted on Whatman 3MM filter papers. Filter papers were air-dried and stored in zip-sealed plastic bags with silica gel absorbent at room temperature until DNA extraction.

### Detecting asymptomatic cases and measuring quantity of parasite DNA

Slides were examined under microscopes at a magnification of 100. Parasites were counted against 200 leukocytes and a slide was considered negative when no parasites were observed after counting over 100 microscopic fields. All slides were read in duplicate by two microscopists at the time of sample collection. For some of the samples that showed discrepancy with the conventional PCR results, we repeated the slide read for verification. The density of parasitemia was expressed as the number of asexual *Plasmodium falciparum* per microliter of blood, assuming a leukocyte count of 8000 per microliter.

Parasite DNA was extracted from dried blood spots by the Saponin/Chelex method [24]. The final extracted volume was 200μl. The presence of *P*. *falciparum* was diagnosed by two PCR assays. DNA from *P*. *falciparum* isolates 7G8 (MRA-926) and HB3 (MRA-155) were used as positive controls in all amplifications, and water and uninfected samples were used as negative control to ensure lack of contamination.

In our pilot study, three different gene regions (18S rRNA, cytochrome, *PFPK*2) were tested on a subset of samples for optimal amplification conditions in conventional PCR and quantitative PCR. We selected gene regions and amplification conditions that produced reproducible results and detected the most positive cases, respectively, in conventional PCR and QPCR to minimize potential bias inherent in the PCR methods. A semi-nested amplification of the *P*. *falciparum-specific* microsatellite *PFPK*2 [25] was finally chosen for parasite detection in all samples. Amplification was conducted in a 20ul reaction mixture containing 2ul of genomic DNA, 10ul of 2×DreamTaq Green PCR Master Mix (Fermentas) and 0.3uM primer. Reaction was performed in BIORAD MyCycler thermal cycler, with an initial denaturation at 94°C for 2 min, followed by 25 cycles at 94°C for 30 sec, 42°C for 30 sec, 40°C for 30 sec, and 65°C for 40 sec, with a final 2 min extension at 65°C in the primary amplification. The secondary amplification was conducted in a 20ul reaction mixture containing 2ul of product from the primary reaction and the same PCR reagents (except for one of the primers) and volume described above. Reaction was performed with an initial denaturation at 94°C for 2 min, followed by 25 cycles at 94°C for 20 sec, 45°C for 20 sec, and 65°C for 30 sec, with a final 2 min extension at 65°C. The amplified products were resolved electrophoretically on a 2% agarose gel in 0.5×Tris-borate (TBE) buffer and visualized under UV light. For samples that showed discrepancy between the microscopic and nested PCR results, we re-extracted DNA from a different blood spot of the same sample and repeated amplifications using the above conditions for verification.

The number of parasite gene copies was compared between the lowland and highland samples, as well as between the microscopic-positive and microscopic-negative samples. A total of 1,168 samples were included in quantitative PCR and these samples were identified positive based on conventional PCR assay (S3 Table). For the lowland sites, 800 out of 1,987 conventional PCR-positive samples were included, of which 447 were microscopic-positive and 353 were microscopic-negative. These samples represented 25 of the 29 lowland sites with a minimum of 10 individuals per site (S3 Table). A larger number of individuals were included for sites with a higher parasite prevalence rate (S1 Table). For the highland sites, all 368 conventional PCR-positive samples from the 18 sites were included in QPCR, of which 128 were microscopic-positive and 240 were microscopic-negative (S3 Table). The number of parasite gene copies, a proxy for parasite density, were estimated using quantitative real-time PCR, specifically the SYBR Green detection method [26] and modified *P*. *falciparum*-specific primers (forward: 5’-AGTCATCTTTCGAGGTGACTTTTAGATTGCT-3’; reverse: 5’- GCCGCAAGCTCCACGCCTGGTGGTGC-3’) that targeted on a falciparum-specific rRNA region. Amplification was conducted in a 20ul reaction mixture containing 2ul of genomic DNA, 10ul 2×SYBR Green qPCR Master Mix (Thermo Scientific), and 0.5uM primer. Reaction was performed in CFX96 Touch Real-Time PCR Detection System (BIORAD), with an initial denaturation at 95°C for 3 min, followed by 45 cycles at 94°C for 30 sec, 55°C for 30 sec, and 68°C for 1 min with a final 95°C for 10 sec. This was then followed by a melting curve step of temperature ranged from 65°C to 95°C with 0.5°C increment to determine the melting temperature of each amplified product. Each assay included both positive controls (7G8 and HB3 isolates) and negative controls (uninfected samples and water). A standard curve was produced from 10-fold dilution series of *P*. *falciparum* control plasmid ranging from 10^5^ to 10^1^ copies/μl to evaluate qPCR amplification efficiency (E). The 10-fold serial dilutions were included in each plate run as internal standard and the amplification efficiency was calculated from the corresponding threshold cycle (*Ct*) values of the dilutions for each plate samples. The amplification efficiency ranges from 90.5–92.8% among all runs. The negative controls showed *Ct* values of above 40. Samples yielding *Ct* values higher than 40 were considered negative for *Plasmodium* species. Melting curve analyses were performed for each amplified sample to confirm specific amplifications of the target sequence. The parasite gene copy number (GCN) in a sample was quantified based on the threshold cycle using the follow equation: GCN_sample_ = 2^E×(40-*Ct*sample)^; where GCN stands for gene copy number, *Ct* for the threshold cycle of the sample, and E for amplification efficiency. Parasite density and gene copy number of samples among sites were reported as geometric mean and range values.

### Statistical analyses

Microsoft Excel software (Microsoft Office 2010) and MedCalc software (version 12; Mariakerke, Belgium) were used for sensitivity and specificity calculations. For all samples (*N* = 11,185), results of conventional PCR were used as the standard. Sensitivity of microscopy was calculated as true positives/(true positive + false negatives), and specificity was calculated as true negatives/(true negatives + false positives). The χ^2^-test was used to test for the significance of differences in parasite prevalence rates by microscopy versus PCR, as well among lowland versus highland sites. One-tailed T-test was used to test for the significance of differences in parasite gene copy number and parasite density among samples of different transmission settings (high versus low transmission sites), and across age groups (under 5, 5–14, and over 14). In addition, we calculated *Pearson's* correlation coefficient (*r*) for continuous data including (1) parasite density based on microscopy and parasite gene copy number based on QPCR; (2) parasite gene copy number and age in R (R Core Team 2013).

## Results

### Comparisons of malaria prevalence by different approaches

**Fig 2:**
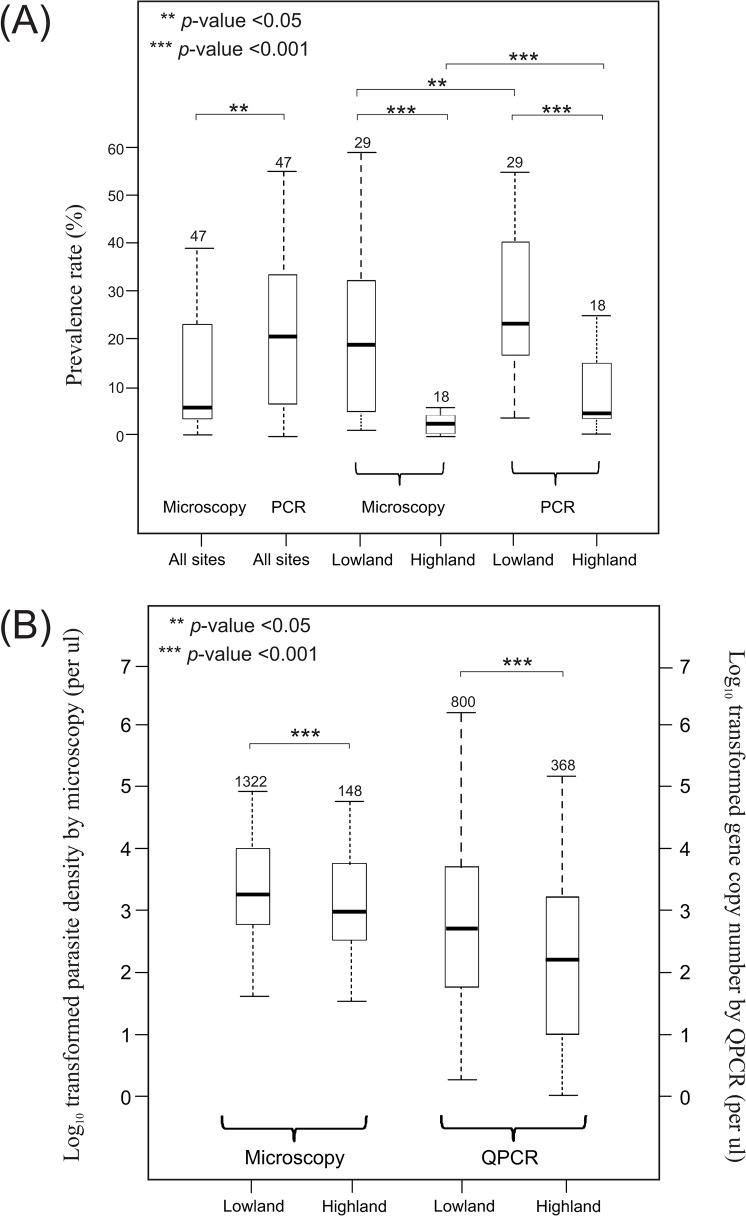
Boxplots comparing (A) prevalence rate detected by microscopy and conventional PCR methods for all sites as well as the lowland and highland sites separately. Numbers above bars indicate number of sites included. Asterisks indicate level of significance; (B) parasitemia level and parasite DNA quantity obtained by microscopy and quantitative polymerase chain reaction (QPCR), respectively, between lowland and highland samples. Numbers above bars indicate number of individuals included. The central box represents the interquartile range and the whiskers represent the first quartile and the fourth quartile of the data. The median is shown as a lien through the center of the box and the ends of the whiskers correspond to the minimum and maximum in the data.

Microscopy and conventional PCR methods revealed substantial variations in malaria prevalence among sites (Fig. 1; S1 Table). Microscopy-based malaria prevalence ranged from 0.4% to 58.2% with an average of 13.3%, and conventional PCR-based prevalence ranged from 2% to 64.7% with an average of 20.9%. Among all sites, 10, 14, and 23 were shown with high (>25%), moderate (5–25%), and low (<5%) prevalence rate by microscopy (Fig. 1A), whereas 21, 18, and 8 sites were shown with high, moderate, and low prevalence rate by conventional PCR (Fig. 1B). Conventional PCR revealed a significantly higher rate of prevalence than microscopy when samples of all sites were pooled together (Fig. 2A). Among the 47 examined sites, 26 of them showed similar and consistent parasite prevalence rate by microscopy and conventional PCR, i.e., these sites were defined as the same prevalence rate category (red, blue, and black color dots in Fig. 1). For the 21 sites that showed discrepancy between the two methods, 17 of them showed more than two-fold higher prevalence rate by conventional PCR than by microscopy; whereas the remaining four sites (KB, PA, SP, and YA) showed slightly higher prevalence rate by microscopy than by conventional PCR. These four sites had 1–7 additional individuals identified by microscopy out of the 200+ examined samples from each site (S1 Table). The contrast in prevalence rate between the two methods remains the same when lowland and highland sites were analyzed separately (Fig. 2A).

Of all 11,185 samples, microscopy detected 1,470 positive cases (13.1%) whereas conventional PCR detected 2,355 cases (21.1%), which was about 8% more of the total samples than microscopy (χ^2^ = 120.88, d.f. = 1, *P*<0.0001; Table 1). When conventional PCR was used as the gold standard, microscopy showed a low sensitivity (50.4%) but high specificity (96.8%; Tables 1 & S2 Table). Interestingly, the discrepancy between the two methods is considerably larger in the highlands where malaria transmission is low. Of the 6,703 samples collected from the lowland sites, microscopy and PCR detected 1,322 (19.7%) and 1,987 (29.6%) positive cases, respectively (χ^2^ = 58.30, d.f. = 1, *P*<0.0001; Table 1). By contrast, of the 4,482 highland samples, PCR detected 368 (8.2%) positive cases, which was nearly three-fold more than those detected by microscopy (148 cases; 3.3%) (χ^2^ = 119.74, d.f. = 1, *P*<0.0001). Sensitivity of microscopy in the highland samples (34.8%) was shown to be 20% lower than in the lowland samples (53.3%), though specificity values of microscopy in both samples were similar (Table 1). Such difference in sensitivity could be explained by the detection of a greater proportion of false negative samples in the highlands than the lowlands (S2 Table).

**Table 1:** Comparison of detective power between microscopy and conventional PCR (microsatellite *PFPK*2 assay) of all blood samples. PCR was used as gold standard for diagnostic measures.

		**PCR method**		**Microscopic method Microscopy vs. PCR**			
		Number of blood samples (%)		Number of blood samples (%)		Diagnostic measure, % (95% CI)	
Samples	*N*	Positive for *P*. *falciparum*	Negative for *P*. *falciparum*	Positive for *P*. *falciparum*	Negative for *P*. *falciparum*	Sensitivity	Specificity
Total	11185	2355 (21.05)	8830 (78.95)	1470 (13.14)	9715 (86.86)	50.40 (48.36–52.44)	96.80 (96.41–97.15)
Lowlands	6703	1987 (29.64)	4716 (70.36)	1322 (19.72)	5381 (80.28)	53.30 (51.07–55.51)	94.42 (93.73–95.06)
Highlands	4482	368 (8.21)	4114 (91.79)	148 (3.30)	4334 (96.70)	34.78 (29.92–39.89)	99.51 (99.25–99.70)

For the 1,470 samples that were detected positive by microscopy, 80% (1,187 out of 1,470) but not all of them were detected positive by conventional PCR assay (S2 Table). Among the 283 microscopic-positive and conventional PCR-negative samples, quantitative PCR indicated positive in 36 (12.7%) of these samples. These 36 samples had an average parasite gene copy number of 1.1×10^1^/μl (range 0.2×10^1^ to 6.7×10^1^/μl), which was apparently lower than those microscopic-positive and conventional PCR-positive samples (geometric mean gene copy number of 6.5×10^3^/μl and range 6.3×10^1^ to 2.6×10^6^/μl). Likewise, the parasite density in the microscopic-positive and conventional PCR-negative samples (geometric mean 3.8×10^2^ parasite/μl and range 4×10^1^ to 3.5×10^3^ parasites/μl) was also found to be lower than the microscopic-positive and conventional PCR-positive samples (mean 8.3×10^3^ parasite/μl and range 4×10^1^ to 1×10^5^ parasites/μl).

### Parasite gene copy number and density between lowlands and highlands

Quantitative PCR and microscopy indicated that parasite gene copy number and density were significantly higher in lowland than in highland samples, albeit with a difference in sample size (Fig. 2B). QPCR revealed a geometric mean gene copy number of 7.9×10^3^/μl and range 1.1×10^1^ to 2.7×10^6^/μl in the lowlands versus a mean of 1.8×10^2^/μl and range 0.08×10^1^ to 2×10^5^/μl in the highlands (*P*<0.001; Fig. 2B; S3 Table). Microscopy indicted a geometric mean of 2.1×10^3^ parasite/μl and range 4×10^1^ to 5.6×10^5^ parasites/μl in the lowlands versus a geometric mean of 1.5×10^2^ parasite/μl and range 1.6×10^1^ to 1.9×10^5^ parasites/μl in the highlands (*P*<0.001; Fig. 2B; S3 Table).

**Fig 3:**
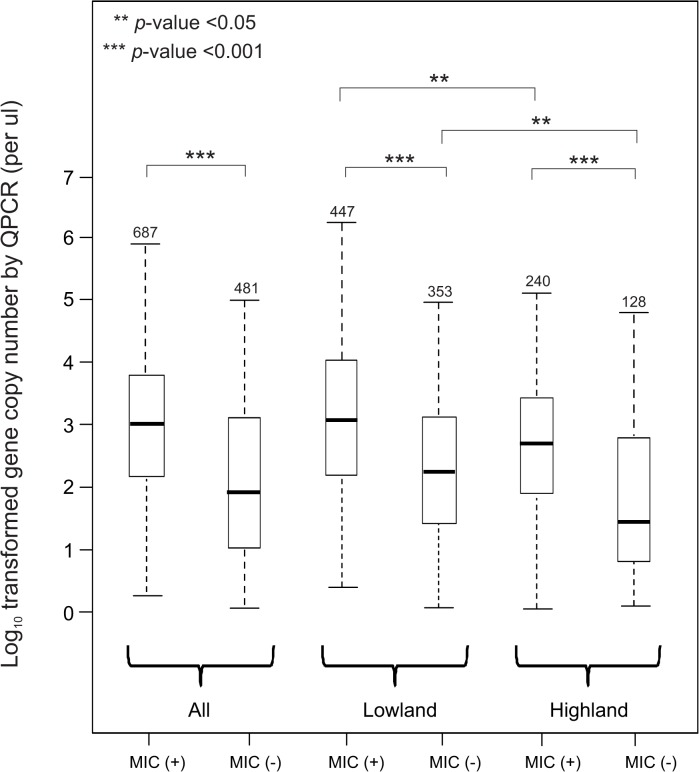
Boxplots showing the amount of parasite DNA detected by SYBR quantitative polymerase chain reaction (QPCR) analysis of subset samples that were diagnosed as positive by conventional PCR. Comparison of parasite DNA quantity was made between (1) microscopy positive and negative samples; and (2) lowland and highland samples. Numbers above bars indicate number of individuals included. The central box represents the interquartile range and the whiskers represent the first quartile and the fourth quartile of the data. The median is shown as a lien through the center of the box and the ends of the whiskers correspond to the minimum and maximum in the data.

The parasite gene copy number in submicroscopic samples (geometric mean 1.0×10^2^/μl and range 0.8×10^1^ to 9.4×10^4^/μl) was found to be significantly lower than in microscopic-positive samples (geometric mean 7.4×10^3^/μl and range 6.3×10^1^ to 2.6×10^6^/μl; *P<*0.001; Fig. 3). This suggested that submicroscopic samples clearly contain parasites, but the parasitemia level could be too low to be detected under microscope. Among the submicroscopic samples, QPCR detected a significantly lower amount of parasite gene copy number in highland than lowland samples (geometric mean gene copy number of 2.1×10^2^/μl and range 2.9×10^1^ to 1.6×10^4^/μl in the lowlands versus geometric mean of 1.2×10^1^/μl and range 0.8×10^1^ to 6.1×10^3^/μl in the highlands; *P* = 0.005; Fig. 3). Such difference in parasite gene copy number was also observed in the microscopic-positive samples between the lowlands (geometric mean 1.1×10^3^/μl and range 1.3×10^1^ to 2.6×10^6^/μ) and highlands (geometric mean 5.7×10^2^/μl and range 0.8×10^1^ to 5.3×10^4^/μ; *P* = 0.03; Fig. 3). Thus, the contrast in parasite gene copy number likely explains the large discrepancy in the sensitivity values between microscopy and conventional PCR when detecting prevalence among highland samples (Table 1). Highland submicroscopic samples in general contain very low level of parasitemia that requires a more sensitive method to detect positivity.

**Fig 4:**
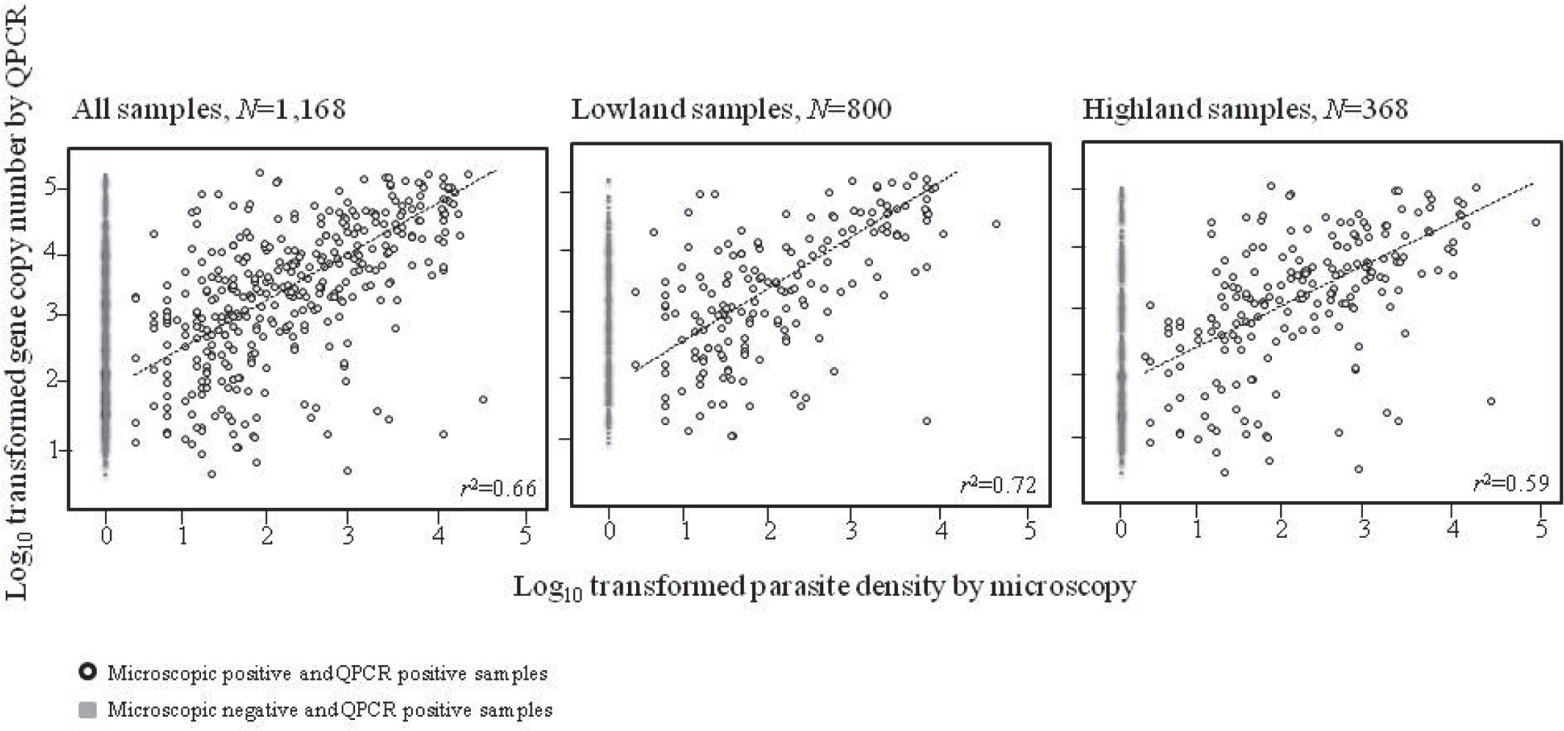
Scatter plot matrix showing correlation of estimates of parasite density obtained by microscopy and parasite DNA quantity by SYBR quantitative polymerase chain reaction (QPCR) analysis of blood samples. Pearson’s product moment (r) correlation coefficients were indicated.

Parasite densities measured by microscopy are significantly correlated with parasite gene copy number by QPCR (*r* ^2^ = 0.66, *P<*0.01; Fig. 4). Such a correlation holds true when samples were analyzed with respect to lowland and highland. The QPCR method indicated a detection limit of above 3.05×10^–5^% parasitemia (equivalent to an estimated 1.5 parasites/mL assuming approximately 5 million red blood cells per microliter of blood) counted on a stained thin blood film (S4 Table).

### Comparison of parasite prevalence and parasitemia with age

**Fig 5:**
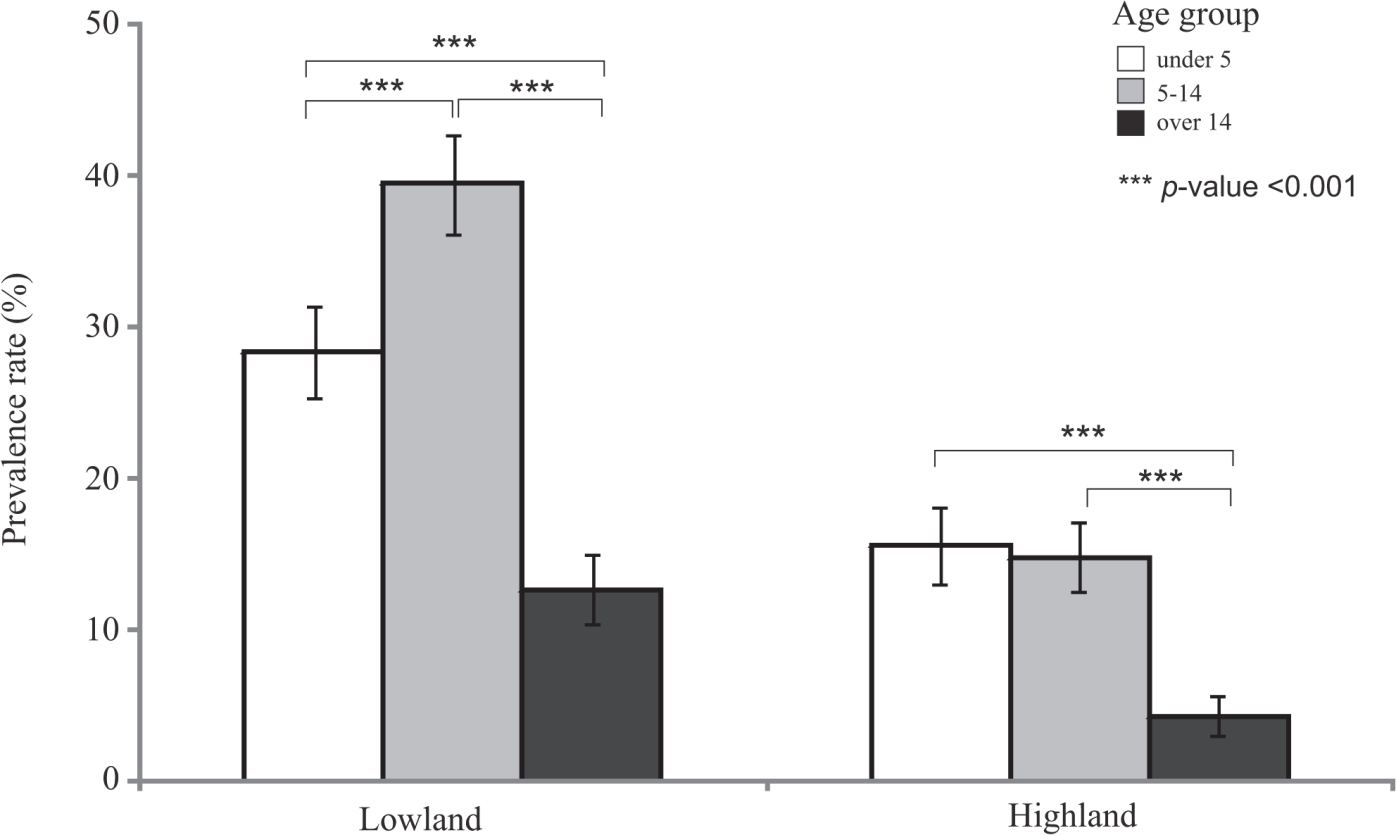
Histogram showing the mean malaria prevalence rate of the three age groups (under 5, aged 5–14, and over 14) in the lowlands and highlands. Error bars indicated the standard deviation of the mean value. The level of significance was indicated.

As expected, parasite prevalence rate was shown to be the lowest in older children (aged over 14) in both highland and lowland sites (Fig. 5). However, in the lowlands the highest prevalence was not found in the youngest children (age under 5) but those of age 5–14. By contrast, in the highlands children of age <5 and 5–14 indicated no significant difference in prevalence rate. The parasite gene copy number did not show a significant correlation with age in both the highland and lowland sites (S1 Fig.). No significance differences were found in gender for both the prevalence rate and parasite gene copy number.

## Discussion

The present study is by far the most comprehensive survey that examines asymptomatic infections in broad areas of Western Kenya and includes a deep sampling at community level based on both microscopy and PCR-based methods. We explore and compare parasite density and gene copy number among sites of different transmission settings as well as across age groups. It is noted that our samples represent only children and adolescents up to 18 years old but not the entire demographic population. This community-based study uncovers the extent of asymptomatic malaria in areas such as the eastern and southern shore of the Lake Victoria where malaria prevalence is previously unknown. The information obtained from this study is important because it provides a basis for identifying priority areas for disease control concerning asymptomatic malaria. Our data indicate that lowland areas in the northern shore of the Lake Victoria have the highest asymptomatic malaria infection rate; and as expected, highland fringe areas show relatively low transmissions. It is, however, noteworthy that not all lowland sites surrounding the lakeshore area have high transmissions, for example, both microscopic and PCR-based malaria prevalence rates were moderate in eastern and southern shore areas. Thus, apart from topography [27], there could be other factors such as mosquito vector composition [28] and socio-economic factors such as human travel, accessibility to health care systems, and effectiveness of control measures that are operating in concert and influence malaria occurrence in the lowlands [29, 30].

Our comparisons of parasite gene copy number among sites using QPCR confirm that submicroscopic asymptomatic infections in the low transmission setting of the highlands have lower level of parasite gene copies than those in the high transmission setting of the lowlands and that such difference impacts diagnostic outcomes. Although the comparison was made on an imbalance sample size given that PCR-positive cases in the lowland are more than six times higher than those in the highland, the samples included in the QPCR assay well represent the majority of the study sites in Western Kenya. The contrast in parasite gene copy number supports the hypothesis that frequent infection in high-transmission areas increase the average parasite density in infected asymptomatic individuals, whereas in lower transmission areas asymptomatic infections are more likely to have reached a submicroscopic phase [6]. These submicroscopic infections remain untreated by current malaria treatment policy and can sustain malaria transmission in areas where malaria endemicity is low [2, 6, 17, 31]. Therefore, improved detection of asymptomatic infections, particularly those of submicroscopic level in local communities, is key to uncover the hidden malaria and to develop new control strategies in order to minimize the burden of malaria.

Our study showed that microscopy detected a significantly smaller number of asymptomatic infections compared to PCR-based method, which is consistent with several previous findings that indicated molecular assay a more sensitive and time-efficient approach for asymptomatic malaria [3, 15, 18, 32–35]. Among the 47 examined sites, 21 of them showed discrepancy between the two detection methods. Seventeen of these sites showed more than two-fold higher prevalence rate by conventional PCR than by microscopy, and four of them showed slightly higher prevalence rate by microscopy than by conventional PCR. Such discrepancies could be due to the shortcomings of the two methods. Microscopy readings often exhibit substantial discordance when compared to molecular diagnosis, producing both false negative (e.g., submicroscopic infections [36–39]) as well as false positive readings (e.g., poor-quality blood films or lack of expertise [36, 40]). The use of microscopy as a ‘gold standard’ has been shown to produce misleading results in clinical trials [41–43]. Several reports have shown that the examination of thin blood smears especially in cases of low parasitemia or mixed infections had lower sensitivity (approximately 50 parasite/μl of blood) than conventional PCR techniques that can detect as few as 1 parasite/μl of blood [40]. Our findings clearly demonstrate that conventional PCR-positive and microscopic-negative samples had a significantly lower level of parasite gene copy number than the PCR-positive and microscopic-positive samples. Thus, it is possible that parasite density in the ‘false negative’ samples is beyond the detection limit of a trained microscopist. However, on the other hand, it is not uncommon for microscopic positive samples to be sometimes detected as PCR negative due to poor DNA quality or mutations in the priming sites of the parasite genome [12, 36]. PCR amplification is a stochastic process and the identification of positivity on an agarose gel can be arbitrary when DNA quality is poor yielding little or weak amplification products and gives a vague band signal. In addition, our data indicate that microscopic-positive and conventional PCR-negative samples showed a considerably lower parasite density than the microscopic-positive and conventional PCR-positive samples. Among the microscopic-positive and conventional PCR-negative samples, QPCR detected a small portion (12.7%) of them as positive and indicated that these samples in general revealed a lower amount of parasite gene copy number compared to the microscopic-positive and conventional PCR-positive samples. Therefore, apart from DNA quality, our data might also suggest that PCR results can sometimes be stochastic when parasite density is low in a sample and this partly explains the discrepancy between microscopy and PCR-based detection methods.

Previous studies found that both uncomplicated clinical malaria and asymptomatic infections peak in young children [7–9] and that young children were most subjected to malaria-diagnosed death [10–11, 44]. Consistent to previous findings, our analyses indicated that children of younger age groups had higher parasite prevalence rate and that the peak age of asymptomatic malaria was found in children of age 5–14. These findings suggested the needs for better age-specific strategies in malaria control programmes and critical evaluations on the effect of existing preventive measures and treatment of children in low and high transmission settings [11, 45, 46]. While parasitaemia would be expected to correlate with age by cumulative exposure to parasite over time in asymptomatic infections or gradual acquisition of immunity, our data did not indicate such a correlation.

In summary, this study highlights the need for a more sensitive and time-efficient assay for asymptomatic malaria in areas of low-transmission settings because of the prevalence of submicroscopic infections with low parasite density and gene copy number. Combining PCR with microscopy can enhance the capacity of detecting low-density asymptomatic malaria infections and allow for an improved characterization of the current reservoir of infections that is largely hidden and heterogeneous. The combined diagnostic approaches are of key importance in providing precise malaria prevalence information for novel surveillance control strategies targeting the most infectious reservoirs.

## Supporting Information

S1 Table Locality information, sampling size, and prevalence rate of each site included in this study. Asterisks indicate sites where prevalence rate detected by microscopy is higher than that by nested PCR.
(DOCX)

S2 Table Number of samples detected by microscopy (MIC) and conventional PCR (PCR) of all blood samples. PCR was used as gold standard.
(DOCX)

S3 Table Mean and range of parasite density measured by microscopy and parasite gene copy number measured by SYBR Green quantitative PCR (QPCR) of *P*. *falciparum* samples from the lowland (elevation below 1,500m) and highland (elevation above 1,500m) sites of Western Kenya. ‘-’ denote data not available. Locality information of sites is presented in S1 Table.
(DOCX)

S4 Table Detection limit of parasitemia based on SYBR Green QPCR method using serial dilutions of *P*. *falciparum* culture. The mean *Ct* values were calculated from three independent runs and standard error values are provided. ‘-’ denote samples not detected by quantitative PCR.
(DOCX)

S1 Fig Scatter plot matrix showing correlation of parasite gene copies by quantitative polymerase chain reaction (QPCR) analysis and age of the studied subjects. Pearson’s product moment (r) correlation coefficients were indicated.
(TIF)

## References

[1] Laishram DD, Sutton PL, Nanda N, Sharma VL, Sobti RC, Carlton JM, et al The complexities of malaria disease manifestations with a focus on asymptomatic malaria. Malaria J 2012;11: 29 10.1186/1475-2875-11-29 22289302 PMC3342920

[2] Karl S, Gurarie D, Zimmerman PA, King CH, St. Pierre TG, Davis TME. A sub-microscopic gametocyte reservoir can sustain malaria transmission. PLoS One 2011;6: e20805 10.1371/journal.pone.0020805 21695129 PMC3114851

[3] Okell LC, Ghani AC, Lyons E, Drakeley CJ. Submicroscopic infection in *Plasmodium falciparum*-endemic populations: a systematic review and meta-analysis. J Infect Dis 2009;200: 1509–1517. 10.1086/644781 19848588

[4] Harris I, Sharrock WW, Bain LM, Gray KA, Bobogare A, Boaz L, et al A large proportion of asymptomatic *Plasmodium* infections with low and sub-microscopic parasite densities in the low transmission setting of Temotu Province, Solomon Islands: challenges for malaria diagnostics in an elimination setting. Malaria J 2010;9: 254 10.1186/1475-2875-9-254 20822506 PMC2944341

[5] Schachterle SE, Mtove G, Levens JP, Clemens EG, Shi L, Raj A, et al Prevalence and density-related concordance of three diagnostic tests for malaria in a region of Tanzania with hypoendemic malaria. J Clin Microbiol 2011;49: 3885 10.1128/JCM.01157-11 21880972 PMC3209109

[6] Okell L, Bousema T, Griffin JT, Ouédraogo AL, Ghani AC, Drakeley CJ. Factors determining the occurrence of submicroscopic malaria infections and their relevance for control. Nat Commun 2012;3: 1237 10.1038/ncomms2241 23212366 PMC3535331

[7] Idro R, Aloyo J, Mayende L, Bitarakwate E, John CC, Kivumbi GW. Severe malaria in children in areas with low, moderate and high transmission intensity in Uganda. Trop Med Int Health 2006;11: 115–24. 16398762 10.1111/j.1365-3156.2005.01518.x

[8] Okiro EA, Al-Taiar A, Reyburn H, Idro R, Berkley JA, Snow RW. Age patterns of severe paediatric malaria and their relationship to *Plasmodium falciparum* transmission intensity. Malaria J 2009;8: 4. 10.1186/1475-2875-8-4 PMC2630996 19128453

[9] Carneiro I, Roca-Feltrer A, Griffin JT, Smith L, Tanner M, Schellenberg JA, et al Age-patterns of malaria vary with severity, transmission intensity and seasonality in Sub-Saharan Africa: a systematic review and pooled analysis. PLoS ONE 2010;5: e8988 10.1371/journal.pone.0008988 20126547 PMC2813874

[10] Munyekenye OG, Githeko AK, Zhou G, Mushinzimana E, Minakawa N, Yan G. *Plasmodium falciparum* Spatial Analysis, Western Kenya Highlands. Emerg Infect Dis 2005;11: 1571–1577. 16318698 10.3201/eid1110.050106 PMC3366738

[11] Atieli HE, Zhou G, Afrane Y, Lee M-C, Mwanzo I, Githeko AK, et al Insecticide-treated net (ITN) ownership, usage, and malaria transmission in the highlands of western Kenya. Parasite Vector 2011;4: 113 10.1186/1756-3305-4-113 21682919 PMC3135563

[12] Coleman RE, Sattabongkot J, Promstaporm S, Maneechai N, Tippayachai B, Kengluecha A, et al Comparison of PCR and microscopy for the detection of asymptomatic malaria in a *Plasmodium falciparum/vivax* endemic area in Thailand. Malaria J 2006;5: 121 17169142 10.1186/1475-2875-5-121 PMC1716172

[13] Gediminas V, Iezhova TA, Križanauskienė A, Palinauskas V, Sehgal RNM, Bensch S. A comparative analysis of microscopy and PCR-based detection methods for blood parasites. J Parasitol 2005;94: 1395–1401. 10.1645/GE-1570.1 18576856

[14] Fançony C, Sebastião YV, Pires JE, Gamboa D, Nery SV. Performance of microscopy and RDTs in the context of a malaria prevalence survey in Angola: a comparison using PCR as the gold standard. Malaria J 2013;12: 284. 10.1186/1475-2875-12-284 PMC3751255 23941281

[15] Khairnar K, Martin D, Lau R, Ralevski F, Pillai DR. Multiples real-time quantitative PCR, microscopy and rapid diagnostic immune-chomatographic tests for the detection of *Plasmodium* spp: performance, limit of detection analysis and quality assurance. Malaria J 2009;8: 284. 10.1186/1475-2875-8-284 PMC2796674 20003199

[16] Pakalapati D, Garg S, Middha S, Kochar A, Subudhi AK, Arunachalam BP, et al Comparative evaluation of microscopy, OptiMAL (R) and 18S rRNA gene based multiplex PCR for detection of Plasmodium falciparum & Plasmodium vivax from field isolates of Bikaner, India. Asian Pac J Trop Med 2013;6: 346–351. 10.1016/S1995-7645(13)60037-1 23608372

[17] Diallo A, Ndam NT, Moussilioous A, Don Santos S, Ndonky A, Borderon M, et al Asymptomatic carriage of *Plasmodium* in urban Dakar: the risk of malaria should not be underestimated. PLoS ONE 2012;7: e31100 10.1371/journal.pone.0031100 22363558 PMC3283586

[18] Bashir IM, Otsyula N, Awinda G, Spring M, Schneider P, Waitumbi JN. Comparison of PfHRP-2/pLDH ELISA, qPCR and microscopy for the detection of *Plasmodium* events and prediction of sick visits during a malaria vaccine study. PLoS ONE 2013;8: e56828 10.1371/journal.pone.0056828 23554856 PMC3598859

[19] Shillcutt S, Morel C, Goodman C, Coleman P, Bell D, Whitty CJM, et al Cost-effectiveness of malaria diagnostic methods in sub-Saharan Africa in an era of combination therapy. Bull World Health Organ 2008;86: 2 18297164 10.2471/BLT.07.042259 PMC2647374

[20] Bejon P, Andrews L, Hunt-Cook A, Sanderson F, Gilbert SC, Hill AVS. Thick blood film examination for *Plasmodium falciparum* malaria has reduced sensitivity and underestimates parasitaemia. Malaria J 2006;5: 104 17092336 10.1186/1475-2875-5-104 PMC1636647

[21] Ochola LB, Vounatsou P, Smith T, Mabaso MLH, Newton CRJC. The reliability of diagnostic techniques in the diagnosis and management of malaria in the absence of a gold standard. Lance Infect Dis 2006;6: 582–588. 16931409 10.1016/S1473-3099(06)70579-5

[22] Farcas GA, Zhong KJ, Mazzulli T, Kain KC. Evaluation of the RealArt malaria LC real-time PCR assay for malaria diagnosis. J Clin Microbiol 2004;42: 636–638. 14766829 10.1128/JCM.42.2.636-638.2004 PMC344507

[23] Perandin F, Manca N, Calderaro A, Piccolo G, Galati L, Ricci L, et al Development of a real-time PCR assay for detection of *Plasmodium falciparum*, *Plasmodium vivax*, and *Plasmodium ovale* for routine clinical diagnosis. J Clin Microbiol 2004;42: 1214–1219. 15004078 10.1128/JCM.42.3.1214-1219.2004 PMC356834

[24] Wooden J, Kyes S, Silbley C. PCR and strain identification in *Plasmodium falciparum* . Parasitol Today 1993;9: 303–5. 15463789 10.1016/0169-4758(93)90131-x

[25] Anderson TJC, Su XZ, Bockarie M, Lagog M, Day KP. Twelve microsatellite markers for characterization of *Plasmodium falciparum* from finger-prick blood samples. Parasitology 1999;119: 113–125. 10466118 10.1017/s0031182099004552

[26] Rougemont M, Van SM, Sahli R, Hinrikson HP, Bille J, Jaton K. Detection of four *Plasmodium* species in blood from humans by 18S rRNA gene subunit-based and species-specific real-time PCR assays. J Clin Microbiol 2004;42: 5636–5643. 15583293 10.1128/JCM.42.12.5636-5643.2004 PMC535226

[27] Jelinek T, Proll S, Hess F, Kabagambe G, Von Sonnenburg E, Loscher T, et al Geographic differences in the sensitivity of a polymerase chain reaction for the detection of *Plasmodium falciparum* infection. Am J Trop Med Hyg 1996;55: 647–651. 9025692 10.4269/ajtmh.1996.55.647

[28] Minakawa N, Dida GO, Sonye GO, Futami K, Njenga SM. Malaria Vectors in Lake Victoria and Adjacent Habitats in Western Kenya. PLoS ONE 2012;7: e32725 10.1371/journal.pone.0032725 22412913 PMC3297610

[29] Shanks GD, Hay SI, Omumbo JA, Snow RW. Malaria in Kenya's western highlands. Emerg Infect Dis 2005;11: 1425–1432. 16229773 10.3201/eid1109.041131 PMC3310610

[30] Martens P, Hall L. Malaria on the move: human population movement and malaria transmission. Emerg Infect Dis 2000;6: 103–109. 10756143 10.3201/eid0602.000202 PMC2640853

[31] Ouedraogo AL, Bousema T, Schneider P, de Vlas SJ, Ilboudo-Sanogo E, Cuzin-Ouattara N, et al Substantial contribution of submicroscopical *Plasmodium falciparum* gametocyte carriage to the infectious reservoir in an area of seasonal transmission. PLoS One 2009;4: e8410 10.1371/journal.pone.0008410 20027314 PMC2793432

[32] Boonma P, Christensen PR, Suwanarusk R, Price RN, Russell B, Lek-Uthai U. Comparison of three molecular methods for the detection and speciation of Plasmodium vivax and Plasmodium falciparum. Malaria J 2007;6: 124 17868467 10.1186/1475-2875-6-124 PMC2020467

[33] Morassin B, Fabre R, Berry A, Magnaval JF. One year's experience with the polymerase chain reaction as a routine method for the diagnosis of imported malaria. Am J Trop Med Hyg 2002;66: 503–508. 12201583 10.4269/ajtmh.2002.66.503

[34] Snounou G, Viriyakosol S, Jarra W, Thaithong S, Brown KN. Identification of the four human malaria parasite species in field samples by the polymerase chain reaction and detection of a high prevalence of mixed infections. Mol Biochem Parasitol 1993;58: 283–292. 8479452 10.1016/0166-6851(93)90050-8

[35] Manjurano A, Okell L, Lukindo T, Reyburn H, Olomi R, Roper C, et al Association of sub-microscopic malaria parasite carriage with transmission intensity in northeastern Tanzania. Malaria J 2011;10: 370 10.1186/1475-2875-10-370 22177014 PMC3276450

[36] Rantala AM, Taylor SM, Trottman PA, Luntamo M, Mbewe B, Maleta K, et al Comparison of real-time PCR and microscopy for malaria parasite detection in Malawian pregnant women. Malaria J 2010;9: 269 10.1186/1475-2875-9-269 20925928 PMC2984567

[37] Mayor A, Moro L, Aguilar R, Bardaji A, Cistero P, Serra-Casas E, et al How hidden can malaria be in pregnant women? Diagnosis by microscopy, placental histology, polymerase chain reaction and detection of histidine-rich protein 2 in plasma. Clin Infect Dis 2012;54: 1561–1568. 10.1093/cid/cis236 22447794

[38] Arango EM, Samuel R, Agudelo OM, Carmona-Fonseca J, Maestre A, Yanow SK. Molecular detection of malaria at delivery reveals a high frequency of submicroscopic infections and associated placental damage in pregnant women from Northwest Colombia. Am J Trop Med Hyg 2013;89: 178–183. 10.4269/ajtmh.12-0669 23716408 PMC3748479

[39] Kaisar MMM, Supali T, Wiria AE, Hamid F, Wammes LJ, Sartono E, et al Epidemiology of *Plasmodium* infections in Flores Island, Indonesia using real-time PCR. Malaria J 2013;12: 169 10.1186/1475-2875-12-169 23706132 PMC3679745

[40] Ohrt C, Sutamihardja MA, Tang D, Kain KC. Impact of microscopy error on estimates of protective efficacy in malaria-prevention trials. J Infect Dis 2002;186: 540–546. 12195382 10.1086/341938

[41] Perandin F, Manca N, Calderaro A, Piccolo G, Galati L, Ricci L, et al Development of a real-time PCR assay for detection of *Plasmodium falciparum*, *Plasmodium vivax*, and *Plasmodium ovale* for routine clinical diagnosis. J Clin Microviol 2004;42: 1214–9. 15004078 10.1128/JCM.42.3.1214-1219.2004 PMC356834

[42] Boonma P, Christensen PR, Suwanarusk R, Price RN, Russell B, Lek-Uthai U. Comparison of three molecular methods for the detection and speciation of *Plasmodium vivax* and *Plasmodium falciparum* . Malaria J 2007;6: 124 17868467 10.1186/1475-2875-6-124 PMC2020467

[43] Mens P, Spieker N, Omar S, Heijnen M, Schallig H, Kager PA. Is molecular biology the best alternative for diagnosis of malaria to microscopy? A comparison between microscopy, antigen detection and molecular tests in rural Kenya and Urban Tanzania. Trop Med Int Health 2007;12: 238–244. 17300631 10.1111/j.1365-3156.2006.01779.x

[44] O’Meara W, Bejon P, Mwangi TW, Okiro EA, Peshu N, Snow RW, et al Effect of a fall in malaria transmission on morbidity and mortality on Kilifi, Kenya. The Lancet 2008;372: 1555–1562. 10.1016/S0140-6736(08)61655-4 18984188 PMC2607008

[45] Greenwood B. Review: Intermittent preventive treatment—a new approach to the prevention of malaria in children in areas with seasonal malaria transmission. Trop Med Int Health 2008;11: 983–91. 10.1111/j.1365-3156.2006.01657.x 16827699

[46] Grobusch MP, Leli B, Schwarz NG, Gabor J, Dornemann J, Potschke M, et al Intermittent preventive treatment against malaria in infeants in Gabon—a randomized, double-blind, placebo-controlled trial. J Infect Dis 2007;196: 1595–602. 18008242 10.1086/522160

