# Supplementary material for: Low Parasitemia in Submicroscopic Infections Significantly Impacts Malaria Diagnostic Sensitivity in the Highlands of Western Kenya
Source: PLoS One. 2015 Mar 27;10(3):e0121763. doi: 10.1371/journal.pone.0121763 (PMC4376713; doi:10.1371/journal.pone.0121763)
Supplement: S1 Table — Asterisks indicate sites where prevalence rate detected by microscopy is higher than that by nested PCR. (DOCX) [file pone.0121763.s001.docx]

**Table S1**.Locality information, sampling size, and prevalence rate of each site included in this study. Asterisks indicate sites where prevalence rate detected by microscopy is higher than that by nested PCR.

| **Locality** | **Site label** | **Elevation (m)** | **Latitude** | **Longitude** | **Vegetation type** | **No. of samples (positive by**  **nestedPCR)** | **Prevalence rate (%)** | **No. of samples (positive by microscopy)** | **Prevalence rate (%)** |
| --- | --- | --- | --- | --- | --- | --- | --- | --- | --- |
| Akala | AK | 1219 | -0.06 | 34.42 | Dryland Cropland and Pasture | 201 (130) | 64.7 | 201 (117) | 58.2 |
| Amkura | AR | 1172 | 0.57 | 34.27 | Shrubland | 201 (28) | 13.9 | 201 (4) | 2.0 |
| Bomet | BM | 2134 | -0.78 | 35.34 | Savanna | 250 (7) | 2.8 | 250 (1) | 0.4 |
| Boro | BO | 1179 | 0.09 | 34.24 | Dryland Cropland and Pasture | 234 (91) | 38.9 | 234 (34) | 14.5 |
| Busia | BU | 1219 | 0.45 | 34.12 | Evergreen Broadleaf Forest | 213 (116) | 54.4 | 216 (84) | 38.9 |
| Chamasire | CM | 1296 | 0.74 | 34.39 | Savanna | 200 (41) | 20.5 | 202 (8) | 4.0 |
| Chulaimbo | CH | 1398 | -0.04 | 34.64 | Cropland/Woodland Mosaic | 189 (65) | 34.4 | 190 (45) | 23.7 |
| Chwele | CW | 1616 | 0.74 | 34.58 | Shrubland | 209 (85) | 40.7 | 208 (29) | 13.9 |
| Eldoret | EL | 2149 | 0.54 | 35.27 | Grassland | 322 (15) | 4.7 | 357 (8) | 2.2 |
| Emutete | EM | 1587 | 0.04 | 34.65 | Cropland/Woodland Mosaic | 236 (59) | 25.0 | - | 9.8 |
| Homa Bay | HB | 1219 | -0.53 | 34.46 | Savanna | 300 (16) | 5.3 | 300 (14) | 4.7 |
| Iguhu | IG | 1522 | 0.16 | 34.75 | Dryland Cropland and Pasture | 187 (87) | 15.0 | - | 5.5 |
| Kabula | KA | 1263 | 0.49 | 34.53 | Shrubland | 222 (109) | 49.1 | 221 (43) | 19.5 |
| Kaimosi | KS | 1529 | 0.13 | 34.85 | Dryland Cropland and Pasture | 161 (35) | 21.7 | 205 (7) | 3.4 |
| Kamajo | AW | 1196 | -0.08 | 34.21 | Shrubland | 181 (46) | 25.4 | 179 (36) | 20.1 |
| Kamkuywa | KM | 1598 | 0.78 | 34.79 | Savanna | 401 (21) | 5.2 | 401 (10) | 2.5 |
| Kanyawegi | KW | 1219 | -0.11 | 34.61 | Shrubland | 217 (82) | 40.1 | 215 (67) | 31.2 |
| Kapsabet | KP | 1900 | 0.20 | 35.12 | Dryland Cropland and Pasture | 369 (32) | 8.7 | 460 (14) | 3.0 |
| Kendu Bay* | KB | 1178 | -0.37 | 34.65 | Savanna | 244 (21) | 8.6 | 251 (27) | 10.7 |
| Kericho | KR | 2142 | -0.37 | 35.28 | Dryland Cropland and Pasture | 197 (8) | 4.1 | 188 (7) | 3.7 |
| Keroka | KK | 2134 | -0.78 | 34.95 | Dryland Cropland and Pasture | 250 (9) | 3.6 | 250 (1) | 0.4 |
| Kilgoris | KG | 1829 | -1.01 | 34.88 | Cropland/Woodland Mosaic | 204 (4) | 2.0 | 204 (1) | 0.5 |
| Kitale | KT | 1857 | 1.02 | 35.00 | Urban or Built-Up Land | 382 (16) | 4.2 | 378 (3) | 0.8 |
| lPali* | PA | 1467 | 0.05 | 34.58 | Cropland/Woodland Mosaic | 217 (45) | 20.7 | 217 (48) | 22.1 |
| Luanda | LD | 1262 | -0.81 | 34.22 | Savanna | 237 (27) | 11.4 | 245 (4) | 1.6 |
| Lugulu | LU | 1198 | 0.39 | 34.30 | Evergreen Broadleaf Forest | 230 (78) | 33.9 | 230 (3) | 1.3 |
| Malava | MA | 1575 | 0.45 | 34.85 | Cropland/Woodland Mosaic | 227 (28) | 12.3 | 229 (3) | 1.3 |
| Marindi | MI | 1213 | -1.07 | 34.47 | Shrubland | 225 (39) | 17.3 | 226 (20) | 8.9 |
| Mayanja | MY | 1514 | 0.65 | 34.52 | Cropland/Woodland Mosaic | 218 (73) | 33.5 | 218 (9) | 4.1 |
| Miwanii | MW | 1210 | -0.06 | 34.98 | Cropland/Woodland Mosaic | 135 (60) | 44.4 | 58 (5) | 8.6 |
| Mukhobola | MU | 1150 | 0.09 | 34.16 | Shrubland | 398 (142) | 35.7 | 398 (134) | 33.7 |
| Mwihila | MH | 1384 | 0.18 | 34.62 | Dryland Cropland and Pasture | 206 (45) | 21.8 | 207 (8) | 3.9 |
| Ngiya | NG | 1232 | 0.04 | 34.37 | Dryland Cropland and Pasture | 226 (100) | 44.2 | 226 (40) | 17.7 |
| Nyamira | NM | 1860 | -0.56 | 34.93 | Savanna | 247 (2) | 0.8 | 250 (2) | 0.8 |
| Oseiko | OS | 1136 | -0.03 | 34.02 | Shrubland | 237 (55) | 23.2 | 237 (51) | 21.5 |
| Paulo | PL | 1410 | -0.77 | 34.60 | Broadleaf Deciduous Forest | 189 (17) | 9 | 201 (2) | 1.0 |
| Port Victoria | VI | 1138 | 0.10 | 33.97 | Shrubland | 593 (167) | 28.2 | 593 (163) | 27.5 |
| Ruambwa | RW | 1144 | 0.13 | 34.09 | Shrubland | 211 (75) | 33.9 | 219 (71) | 32.4 |
| Sarora | SR | 1777 | 0.46 | 35.00 | Savanna | 267 (17) | 6.4 | 285 (8) | 2.8 |
| Sega | SE | 1178 | 0.25 | 34.23 | Shrubland | 221 (58) | 26.2 | 221 (59) | 26.7 |
| Shitsitswi | ST | 1246 | 0.26 | 34.51 | Dryland Cropland and Pasture | 223 (112) | 50.2 | 224 (8) | 3.6 |
| Sikubale | SB | 1294 | 0.48 | 34.68 | Cropland/Woodland Mosaic | 133 (22) | 16.5 | 133 (16) | 12.0 |
| Simenya | SI | 1201 | 0.15 | 34.36 | Shrubland | 254 (104) | 40.9 | 254 (83) | 32.7 |
| Sio Port* | SP | 1130 | 0.22 | 34.02 | Shrubland | 210 (59) | 28.1 | 210 (66) | 31.4 |
| Soy | SO | 1733 | 0.68 | 35.14 | Grassland | 195 (29) | 14.9 | 198 (1) | 0.5 |
| Webuye | WE | 1550 | 0.61 | 34.76 | Savanna | 416 (17) | 4.1 | 416 (1) | 0.2 |
| Yala* | YA | 1448 | 0.10 | 34.54 | Cropland/Woodland Mosaic | 205 (61) | 29.8 | 205 (62) | 30.2 |
