# Supplementary material for: Low Parasitemia in Submicroscopic Infections Significantly Impacts Malaria Diagnostic Sensitivity in the Highlands of Western Kenya
Source: PLoS One. 2015 Mar 27;10(3):e0121763. doi: 10.1371/journal.pone.0121763 (PMC4376713; doi:10.1371/journal.pone.0121763)
Supplement: S2 Table — PCR was used as gold standard. (DOCX) [file pone.0121763.s002.docx]

**Table S2**.Number of samples detected by microscopy (MIC) and conventional PCR (PCR) of all blood samples. PCR was used as gold standard.

|  | *N* | PCR+ | PCR- | MIC+ | MIC- | PCR+  MIC+ | PCR+  MIC- | PCR-  MIC+ | PCR- MIC- |
| --- | --- | --- | --- | --- | --- | --- | --- | --- | --- |
| **Total** | 11185 | 2355 | 8830 | 1470 | 9715 | 1187 | 1168 | 283 | 8547 |
| **Lowlands** | 6703 | 1987 | 4716 | 1322 | 5381 | 1059 | 928 | 263 | 4453 |
| **Highlands** | 4482 | 368 | 4114 | 148 | 4334 | 128 | 240 | 20 | 4094 |
