# Supplementary material for: Low Parasitemia in Submicroscopic Infections Significantly Impacts Malaria Diagnostic Sensitivity in the Highlands of Western Kenya
Source: PLoS One. 2015 Mar 27;10(3):e0121763. doi: 10.1371/journal.pone.0121763 (PMC4376713; doi:10.1371/journal.pone.0121763)
Supplement: S3 Table — ‘-’ denote data not available. Locality information of sites is presented in S1 Table. (DOCX) [file pone.0121763.s003.docx]

**Table S3**.Geometric mean and range of parasite density measured by microscopy and parasite gene copy number measured by SYBR Green quantitative PCR (QPCR) of *P. falciparum* samples from the lowland (elevation below 1,500m) and highland (elevation above 1,500m) sites of Western Kenya. ‘-‘ denote data not available. Locality information of sites is presented in Table S1.

| **Locality setting** | **Site** | **Site label** | **Elevation (m)** | **Sample size** | **Geometric mean gene copy number per μl by QPCR (range)** | **Sample size** | **Geometric mean parasite density**  **perμl by microscopy (range)** |
| --- | --- | --- | --- | --- | --- | --- | --- |
| Lowland |  |  |  |  |  |  |  |
|  | Akala | AK | 1219 | 37 | 3.4×10^2^  (3.4×10^1^-5.4×10^4^) | 117 | 2.6×10^3^  (1.2×10^2^-1.2×10^5^) |
|  | Amkura | AR | 1172 | 10 | 1.0×10^5^  (1.3×10^4^-2.7×10^5^) | 4 | 6.5×10^4^  (3.5×10^4^-8.2×10^4^) |
|  | Boro | BO | 1179 | 30 | 9.3×10^2^  (4×10^2^-1.8×10^4^) | 34 | 1.5×10^3^  (1.2×10^2^-3.4×10^4^) |
|  | Busia | BU | 1219 | 122 | 5.0×10^2^  (2.5×10^1^-1.3×10^4^) | 84 | 1.4×10^3^  (1.2×10^2^-6.2×10^4^) |
|  | Chamasire | CM | 1296 | 16 | 1.8×10^3^  (4.6×10^2^-3.9×10^5^) | 8 | 1.1×10^3^  (4×10^1^-4.1×10^5^) |
|  | Chulaimbo | CH | 1398 | 54 | 4.5×10^3^  (1.9×10^2^-1.6×10^5^) | 45 | 9.9×10^2^  (1.6×10^2^-8.9×10^4^) |
|  | Homa Bay | HB | 1219 | 31 | 8.7×10^3^  (4.1×10^1^-1.3×10^5^) | 14 | 3.9×10^3^  (3.6×10^2^-8×10^4^) |
|  | lPali | PA | 1467 | - | - | 48 | 9.6×10^2^  (4×10^1^-3.6×10^4^) |
|  | Kabula | KA | 1263 | - | - | 43 | 2.7×10^3^  (2.8×10^2^-2.4×10^4^) |
|  | Kamajo | AW | 1196 | 44 | 1.1×10^2^  (4.7×10^1^-9.7×10^3^) | 36 | 2.3×10^3^  (3.6×10^2^-1.2×10^5^) |
|  | Kanyawegi | KW | 1219 | - | - | 67 | 2.1×10^3^  (2×10^2^-5.6×10^5^) |
|  | Kendu Bay | KB | 1178 | 28 | 1.8×10^3^  (1.6×10^1^-3.4×10^5^) | 27 | 2.2×10^4^  (4×10^2^-1.8×10^5^) |
|  | Luanda | LD | 1262 | 27 | 7.4×10^2^  (7.5×10^2^-8.5×10^5^) | 4 | 1.5×10^4^  (3.2×10^2^-4×10^4^) |
|  | Lugulu | LU | 1198 | 15 | 6.8×10^3^  (2.6×10^2^-1.6×10^6^) | 3 | 1.1×10^4^  (1.4×10^3^-4.8×10^5^) |
|  | Marindi | MI | 1213 | 45 | 3.8×10^3^  (2.1×10^2^-5.3×10^5^) | 20 | 1.5×10^4^  (1.6×10^2^-2.3×10^5^) |
|  | Miwanii | MW | 1210 | 10 | 2.8×10^4^  (1.9×10^3^-5.7×10^5^) | 5 | 6.6×10^5^  (2.5×10^4^-3.6×10^5^) |
|  | Mukhobola | MU | 1150 | 35 | 7.2×10^3^  (1.7×10^1^-4.2×10^5^) | 134 | 9.8×10^3^  (1.2×10^2^-3.9×10^5^) |
|  | Mwihila | MH | 1384 | - | - | 8 | 1.8×10^4^  (5.2×10^2^-8.4×10^4^) |
|  | Ngiya | NG | 1232 | - | - | 40 | 1.0×10^3^  (3.2×10^2^-2×10^4^) |
|  | Oseiko | OS | 1136 | 20 | 1.6×10^2^  (5.8×10^1^-4.9×10^3^) | 51 | 2.5×10^3^  (1.6×10^2^-1.7×10^5^) |
|  | Paulo | PL | 1410 | 17 | 1.4×10^3^  (7.5×10^1^-5.4×10^5^) | 2 | 5.7×10^4^  (7.2×10^2^-9.4×10^4^) |
|  | Port Victoria | VI | 1138 | 34 | 1.2×10^3^  (1.1×10^1^-2×10^5^) | 163 | 1.2×10^4^  (2×10^2^-2×10^5^) |
|  | Ruambwa | RW | 1144 | 30 | 2.0×10^3^  (2.1×10^1^-5.7×10^4^) | 71 | 1.8×10^3^  (2.8×10^2^-1.8×10^4^) |
|  | Sega | SE | 1178 | 39 | 2.8×10^3^  (7.2×10^1^-1.4×10^6^) | 59 | 1.1×10^3^  (8×10^1^-3.2×10^5^) |
|  | Shitsitswi | ST | 1246 | 63 | 2.8×10^3^  (1.2×10^2^-6.5×10^5^) | 8 | 3.3×10^3^  (4.4×10^2^-2.8×10^4^) |
|  | Sikubale | SB | 1294 | 19 | 1.4×10^3^  (3.2×10^1^-2.7×10^6^) | 16 | 1.8×10^3^  (4×10^2^-5.3×10^5^) |
|  | Simenya | SI | 1201 | 15 | 4.8×10^2^  (4.5×10^1^-2.9×10^3^) | 83 | 2.8×10^3^  (4×10^1^-4.7×10^5^) |
|  | Sio Port | SP | 1130 | 22 | 3.3×10^3^  (4×10^2^-6.6×10^4^) | 66 | 1.5×10^3^  (1.6×10^2^-2×10^5^) |
|  | Yala | YA | 1448 | 17 | 4.1×10^2^  (9.4×10^1^-4.1×10^4^) | 62 | 1.2×10^3^  (8×10^1^-1.6×10^4^) |
|  |  |  | **Total** | **800** | **Total** | **1322** |  |
| Highland |  |  |  |  |  |  |  |
|  | Bomet | BM | 2134 | 7 | 0.8×10^1^  (0.3×10^1^-8.7×10^2^) | 1 | 2.4×10^3^ |
|  | Chwele | CW | 1616 | 36 | 6.1×10^2^  (0.9×10^1^-2×10^5^) | 29 | 2.3×10^3^  (4×10^1^-1.5×10^5^) |
|  | Eldoret | EL | 2149 | 15 | 3.7×10^1^  (0.8×10^1^-2×10^5^) | 8 | 3.6×10^2^  (1.6×10^1^-7.2×10^2^) |
|  | Emutete | EM | 1587 | 20 | 2.7×10^2^  (0.8×10^1^-1.2×10^4^) | 20 | 2.0×10^3^  (1.6×10^2^-5.9×10^4^) |
|  | Iguhu | IG | 1522 | 30 | 6.2×10^2^  (0.9×10^1^-2.1×10^4^) | 23 | 2.7×10^3^  (4×10^1^-9.1×10^4^) |
|  | Kaimosi | KS | 1529 | 35 | 3.3×10^2^  (1.1×10^1^-5.2×10^4^) | 7 | 1.6×10^3^  (3.6×10^2^-2.6×10^4^) |
|  | Kamkuywa | KM | 1598 | 21 | 1.4×10^2^  (0.8×10^1^-2.1×10^4^) | 10 | 2.8×10^3^  (1.2×10^2^-8×10^4^) |
|  | Kapsabet | KP | 1900 | 32 | 0.9×10^1^  (0.6×10^1^-1.1×10^3^) | 14 | 9.9×10^2^  (8×10^1^-3.1×10^4^) |
|  | Kericho | KR | 2142 | 8 | 1.4×10^2^  (0.3×10^1^-7.3×10^3^) | 7 | 3.9×10^4^  (3.6×10^2^-1.9×10^5^) |
|  | Keroka | KK | 2134 | 9 | 0.1×10^1^  (0.2×10^1^-8.5×10^1^) | 1 | 8×10^1^ |
|  | Kilgoris | KG | 1829 | 4 | 2.0×10^1^  (0.8×10^1^-5.5×10^4^) | 1 | 7.2×10^2^ |
|  | Kitale | KT | 1857 | 16 | 4.3×10^2^  (0.3×10^1^-7.6×10^3^) | 3 | 1.3×10^2^  (8×10^1^-2.4×10^2^) |
|  | Malava | MA | 1575 | 28 | 1.1×10^2^  (0.2×10^1^-1.1×10^5^) | 3 | 1.8×10^4^  (8.5×10^3^-3.7×10^4^) |
|  | Mayanja | MY | 1514 | 42 | 5.0×10^2^  (3.7×10^1^-8.2×10^4^) | 9 | 6.2×10^3^  (5.2×10^2^-1×10^5^) |
|  | Nyamira | NM | 1860 | 2 | 8.7×10^1^  (0.08×10^1^-1.9×10^3^) | 2 | 1.0×10^3^  (3.5×10^2^-3.2×10^4^) |
|  | Sarora | SR | 1777 | 17 | 1.6×10^1^  (0.07×10^1^-1.7×10^2^) | 8 | 4.7×10^2^  (3.6×10^2^-5.3×10^3^) |
|  | Soy | SO | 1733 | 29 | 0.9×10^1^  (0.3×10^1^-3.1×10^4^) | 1 | 1.3×10^4^ |
|  | Webuye | WE | 1550 | 17 | 9.1×10^2^  (0.2×10^1^-5×10^4^) | 1 | 9.4×10^3^ |
|  |  |  | **Total** | **368** | **Total** | **148** |  |
