# Supplementary material for: Low Parasitemia in Submicroscopic Infections Significantly Impacts Malaria Diagnostic Sensitivity in the Highlands of Western Kenya
Source: PLoS One. 2015 Mar 27;10(3):e0121763. doi: 10.1371/journal.pone.0121763 (PMC4376713; doi:10.1371/journal.pone.0121763)
Supplement: S4 Table — The mean Ct values were calculated from three independent runs and standard error values are provided. ‘-’ denote samples not detected by quantitative PCR. (DOCX) [file pone.0121763.s004.docx]

**Table S4**.Detection limit of parasitemia based on SYBR Green QPCR method using serial dilutions of *P. falciparum* culture. The mean *Ct* values were calculated from three independent runs and standard error values are provided. ‘-‘ denotes samples not detected by quantitative PCR.

| **Parasitemia** | **Mean *Ct* values** |
| --- | --- |
| 1 % | 26.74 ± 0.19 |
| 0.125% | 27.94± 0.11 |
| 1.56×10^-2^ % | 30.97± 0.18 |
| 1.95×10^-3^ % | 34.25± 0.21 |
| 2.44×10^-4^ % | 37.87± 0.27 |
| 3.05×10^-5^ % | 40.69± 0.42 |
| 3.80×10^-6^ % | - |
| 1.90×10^-6^ % | - |
| 9.50×10^-7^ % | - |
