# Supplementary figures and images for: Low Parasitemia in Submicroscopic Infections Significantly Impacts Malaria Diagnostic Sensitivity in the Highlands of Western Kenya
Source: PLoS One. 2015 Mar 27;10(3):e0121763. doi: 10.1371/journal.pone.0121763 (PMC4376713; doi:10.1371/journal.pone.0121763)

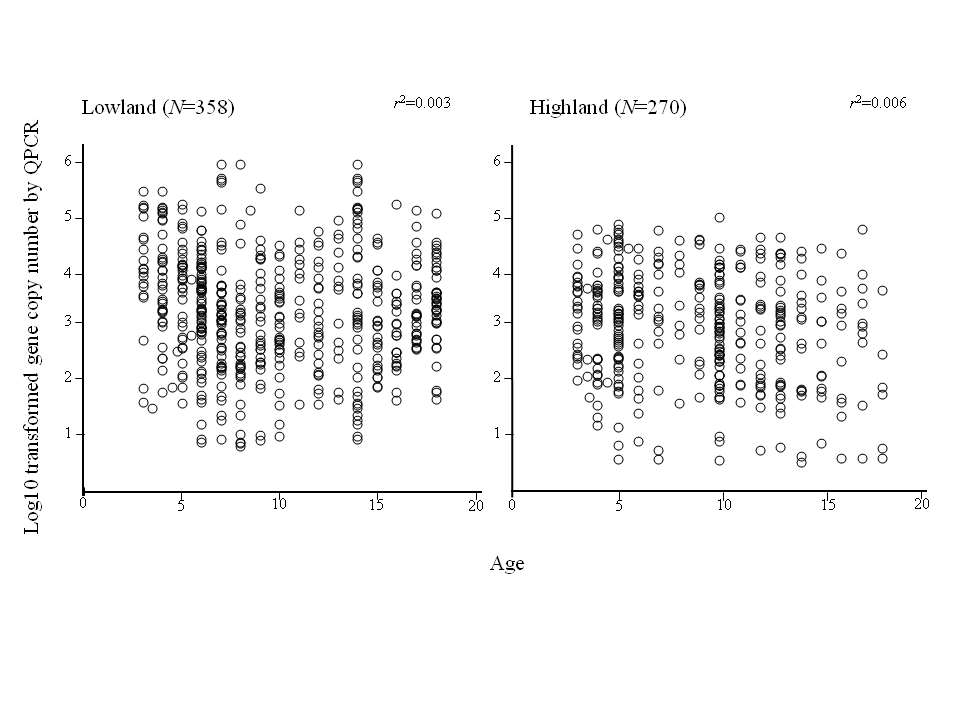

Supplement: S1 Fig — Pearson’s product moment (r) correlation coefficients were indicated. (TIF) [file pone.0121763.s005.tif]
